# Real-Time Optical Monitoring of Pt Catalyst Under the Potentiodynamic Conditions

**DOI:** 10.1038/srep38847

**Published:** 2016-12-09

**Authors:** Hyeon Don Song, Minzae Lee, Gil-Pyo Kim, Inhee Choi, Jongheop Yi

**Affiliations:** 1 World Class University (WCU) Program of Chemical Convergence for Energy & Environment (C2E2), School of Chemical and Biological Engineering, Seoul National University, Gwanak-ku, Seoul 151-742, Republic of Korea; 2 Department of Life Science, University of Seoul, Dongdaemun-ku, Seoul 130-743, Republic of Korea

## Abstract

*In situ* monitoring of electrode materials reveals detailed physicochemical transition in electrochemical device. The key challenge is to explore the localized features of electrode surfaces, since the performance of an electrochemical device is determined by the summation of local architecture of the electrode material. Adaptive *in situ* techniques have been developed for numerous investigations; however, they require restricted measurement environments and provide limited information, which has impeded their widespread application. In this study, we realised an optics-based electrochemical *in situ* monitoring system by combining a dark-field micro/spectroscopy with an electrochemical workstation to investigate the physicochemical behaviours of Pt catalyst. We found that the localized plasmonic trait of a Pt-decorated Au nanoparticle as a model system varied in terms of its intensity and wavelength during the iterations of a cyclic voltammetry test. Furthermore, we show that morphological and compositional changes of the Pt catalyst can be traced in real time using changes in quantified plasmonic characteristics, which is a distinct advantage over the conventional electrochemistry-based *in situ* monitoring systems. These results indicate the substantial promise of online operando observation in a wide range of electrical energy conversion systems and electrochemical sensing areas.

Exploring the local areas of electrode materials in real time is essential for improving electrochemical cell performance and tailored trouble-shooting. Since conventional electrochemical measurement only provides electrical information (*i.e.*, electric current, voltage, and resistance) about electrode materials, precise observation on the localized level has been limited. The morphologies^1^,^2^,^3^, phases^4^, elemental contents^5^, absorbances^6^, and oxidation states^7^ of electrode materials have been examined during electrochemical reactions using *in situ* devices based on typically available equipment for material characterization. However, restricted measurement conditions (*e.g.*, vacuum)^1^,^2^,^7^, limited information (*e.g.*, 2-D projection^1^,^2^,^3^ and low sensitivity^4^), requirements for specially designed cell-kits^1^,^2^,^3^,^4^,^5^,^6^,^7^, and low scan speed^6^ have hampered their widespread application. Therefore, the main challenge is to develop and assess an easily available *in situ* system that can provide local information about the electrode surface, which would allow fast and quantitative observation with high sensitivity.

A promising approach to develop such a versatile device is to utilise the microspectroscopic technique. The localized surface plasmon resonance (LSPR) method using a dark-field microscope combined with spectroscopy (*i.e.*, dark-field microspectroscopy) for metallic nanoparticles (*e.g.*, Au, Pt, and Ag) has been widely used for the optical sensing of biological and chemical species, and has high sensitivity toward changes in particle size, shape, composition, and their surrounding refractive indexes^8^,^9^. Owing to these characteristics, the use of the LSPR technique with simultaneous electrochemical analysis does have merits in the rapid tracking of morphological changes of metallic nanoparticles on electrodes with high sensitivity.

Herein we show the use of dark-field microspectroscopy with electrochemical workstation, namely electrochemical dark-field microspectroscopic cell (EDMC), enables *in situ* observation of the behaviour of noble metal nanoparticles under potentiodynamic condition in a quantitative manner (Fig. 1a).

In the field of energy conversion, most electrode materials consist of metallic species^10^. Among these, Pt is widely used as an electrocatalytic material owing to its high activity. To date, observing the degradation behaviours of Pt nanoparticles during electrochemical reactions has been considered a significant challenging issue owing to its lack of long-term stability^11^. In general, to clarify the degradation phenomena and causes, X-ray and electron microscopic techniques have been utilised for examining the physical changes of Pt species^12^,^13^. X-ray technique provides bulk properties of electrode material, but is lack of high sensitivity. Electron microscopic technique (*e.g.*, identical location transmission electron microscope) provides local information, but is only restricted to very high magnification for the observation of <10 nm nanoparticles. This magnified local area is too small to represent the overall features of electrode material. More importantly, these systems are still *ex-situ* and/or non-real time systems, which impede the precise observation of the intermediate changes of Pt during the electrochemical reactions^14^. We investigated such behaviours of Pt species in acidic media using our designed EDMC system (Fig. 1b) in real time. The investigation was performed under potentiodynamic conditions, which provide operating environments similar to real electrochemical systems. This combination can be operated under ambient conditions such as atmospheric pressure, and no damage on the electrode material is guaranteed during the measurement. Moreover, this system requires no specially designed complex cell-kit and allows real-time and quantitative analysis of changes in physicochemical properties of metallic nanoparticles (*e.g.*, morphology and composition) via measured plasmonic scattering spectra.

## Results and Discussion

### EDMC Set-up and catalyst

As a proof-of-concept demonstration of our system, a nano-assembly comprising a 45 nm Au “core” particle with Pt “satellite” particles (Au@Pt core-satellite) was tested. Since the scattered light caused by a <10 nm Pt nanoparticle is in the ultraviolet (UV) range^15^, direct observation is not possible for human eyes. Therefore, coupling of such Pt particles with a proper core material that can produce scattered light in the visible-light range (*e.g.*, Au nanoparticles) enables the direct observation of the changes of exterior Pt nanoparticles. Moreover, electrochemical reactivity of Au is negligibly low over the given range of the applied potential, which allows our EDMC system to observe Pt species only (Figure S1 in the Supplementary materials).

The Au@Pt core-satellite was synthesized from a 45 ± 1 nm Au nanoparticle as a core material and Pt satellites were decorated on the Au nanoparticle using a reductive heating method (Fig. 2a)^16^. Pt nanoparticles tend to be grown on the surface of a Au nanoparticle in the presence of a weak reducing agent such as ascorbic acid via the Stranski-Krastanov mode rather than through self-nucleation to form individual Pt nanoparticles^17^,^18^. The morphology of the Au@Pt core-satellite was primarily investigated through HR-TEM (Fig. 2b,c). The elemental mapping shows uniformly decorated Pt satellites layers (~6 nm thick) on the Au core nanoparticle (Fig. 2d). The light absorption wavelength for the 45 nm Au nanoparticle was around 540 nm, whereas no pronounced light absorption was observed for the Au@Pt core-satellite (Figure S2 in the Supplementary materials). This difference in optical properties between them allows our EDMC system to trace the physicochemical properties of Pt in real time.

### *in situ* measurement

The *in situ* experiments were performed during the 400 cycles of the cyclic voltammetry (CVs) tests. Optical responses were obtained at specific potential (0.8 V (vs. Ag/AgCl)) during the cathodic sweep in order to exclude the effect of applied potential (Figure S3 in the Supplementary materials). To observe the changes in Pt behaviours during the CVs, we examined the scattering colour changes for Au@Pt core-satellite nanoparticles (Fig. 3a). The resonant scattering colour was dark green before applying the potential. Upon exposing the Au@Pt core-satellite to the electric potential, the scattered colour becomes brighter as the CV cycle number increases. This indicates that physicochemical changes occurred in the Au@Pt core-satellite as the redox process was repeated. The corresponding resonant scattering spectra of the Au@Pt core-satellite were recorded at around 550 nm (Fig. 3b). It was observed that the peak gradually blue-shifted until the 150^th^ cycle of the CVs at the early stage, whereas the peak red-shifted back after the 200^th^ cycle at the latter stage (Fig. 3c). Moreover, the peak intensity tended to increase as the number of CV cycles increased, which is in good agreement with the increase in the brightness of the scattered colours observed from dark-field scattering images.

The changes in electrical currents were also measured simultaneously (Fig. 3d). Hydrogen underpotential deposition and a stripping region below 0.1 V (vs. Ag/AgCl) were observed, which were followed by oxide formation on Pt at a potential above 0.2 V (vs. Ag/AgCl)^19^. The decrease in electrical current as the CV cycle number increased during the potential sweep from −0.2 to 0.0 V (vs. Ag/AgCl) indicates that the amount of desorbed hydrogen is decreased, which is typically ascribed to the reduction of the Pt surface area^10^. The electrochemically active surface area (ECSA) of the Au@Pt core-satellite was calculated from the integrated area of the hydrogen stripping region, and showed a nearly linear decrease as the CV cycle number increased owing to the gradual deactivation of Pt species (Fig. 3e).

We should note that the changes in optical responses with the number of CV cycle are directly attributed to the changes in physico-chemical property of Pt since the plasmonic properties are strongly influenced by the size, shape, and composition of the target metal nanoparticle^20^,^21^,^22^. To confirm these changes of Pt for each CV cycle, the morphologies of Au@Pt assembly and their compositions were examined using TEM (Fig. 4a) and ICP analyses (Table 1), respectively, in a time-lapse manner. It was observed that the apparent boundaries between Pt particles gradually disappeared and the thickness of the Pt layer decreased at the early stage (<100^th^ cycle). In this step, the dissolution of Pt satellites to Pt ionomers (*e.g.*, Pt^2+^ or Pt^4+^) occurs preferentially even though the redeposition of Pt ionomers to Pt^0^ still takes place, owing to the absence of Pt ionomers in the electrolyte before the beginning of the electrochemical reaction. The loss of Pt satellites causes the decrease in the particle size of the entire Au@Pt core-satellite assembly, which led to the spectral blue-shift (Fig. 3c). After the 100^th^ cycle, it was observed that obvious grain boundaries between neighboring Pt particles mostly disappeared and the size of an entire Au@Pt assembly increased with respect to the cycle number. Assuming that the rate of Pt deposition matches the rate of Pt dissolution, the reductive behaviour of Pt ionomers would be more dominant than that of oxidative one. Based on the results obtained after the 200^th^ cycle, Pt ionomers tend to be reduced by linking the spatial gaps between Pt satellites, forming irregular shell-like Pt layers. The gradual formation of shell-like Pt leads to the increase in the size of an entire Au@Pt assembly, which results in the spectral red-shift after the 100^th^ cycle (Fig. 3c).

### Validation of experimental results *via* simulation

For further confirmation of plasmonic characteristics with respect to the experimentally obtained morphological changes in the Au@Pt assembly (Fig. 4a), optical responses for the probable Au@Pt assemblies were investigated theoretically in a quantitative manner by performing simulations (Figure S4 in the Supplementary materials). To calculate the LSPR scattering spectra of the Au@Pt assemblies, we used the discrete dipole approximation (DDA) method^23^. Spectral simulations were carried out using structural models of both the Au@Pt core-satellite and Au@Pt core-shell. The structural models were determined by the results of mass ratio between Pt and Au through ICP analyses. The results of the simulation showed that the spectral intensity and wavelength for the Au@Pt core-satellite are different from those for the Au@Pt core-shell (Fig. 4b,c). These spectral differences indicate the tunability of the optical response with respect to the morphology and mass ratio of Pt. The experimentally obtained LSPR peak intensity for the 0^th^ cycle (*i.e.*, before applying the potential) was initially located on the plot simulated for the Au@Pt core-satellites. Then, it moved toward the plot for the Au@Pt core-shell (≥200^th^ cycle) with respect to the corresponding Pt/Au mass ratio (*i.e.*, cycle number), indicating that the core-satellite-structured Au@Pt gradually transforms into the core-shell-like structure. In particular, a relatively rapid change in intensity was observed before the 200^th^ cycle and the intensity increased slightly after the 200^th^ cycle, implying that the morphological transformation from Pt satellite to Pt shell predominantly occurs before the 200^th^ cycle. The wavelength shift in the experimentally obtained spectra was also compared with that in the simulated LSPR spectra (Fig. 4c). The experimentally obtained LSPR wavelength for the Au@Pt assembly was blue-shifted (negatively from 0 to −15 nm) from the simulated plot for the core-satellite (0^th^ cycle) to that of the core-shell (<100^th^ cycle), and then red-shifted (positively from −15 to −5 nm). The gap (~8 nm) in the spectral shift after the 100^th^ cycle between the experimentally obtained spectra and the simulated spectra is due to the assumption that the surface of the Pt shell is perfectly smooth in the simulation, whereas the experimentally observed thickness of the Pt shell is uneven.

To examine the suitability of the model structures of the Au@Pt assemblies, we compared the Pt surface area of simulation models (*SA*_Pt-LSPR_) with that from the TEM image (*SA*_Pt-TEM_) with respect to the number of CV cycles (Fig. 4d). The details of the calculation are shown in Figure S5 (Supplementary materials). The value of the order and trend of the decrease in *SA*_Pt-LSPR_ were found to be similar to those of *SA*_Pt-TEM_ until the 100^th^ cycle, implying that our assumption regarding the morphological change of Pt is applicable to the real experimental system. The higher value of *SA*_Pt-TEM_ than that of *SA*_Pt-LSPR_ after the 100^th^ cycle is ascribed to the assumption of the calculation that the overlapped Pt surface area between neighboring Pt satellites for *SA*_Pt-TEM_ was left out of account. This is because TEM only provides a 2D-projection image and this makes distinguishing the exact overlapped area between neighboring Pt satellites difficult. Therefore, the calculation was performed by taking solely the surface area obtained from spherical Pt satellites into account for *SA*_Pt-TEM_. Nevertheless, it should be noted that the decrease in the Pt surface area (Fig. 4d) also shows a similar tendency to the decrease in the ECSA (Fig. 3e), which supports the fact that the decrease in the Pt surface area is one of the main factors for the reduction of ECSAs.

## Conclusions

In summary, we demonstrate *in situ* optical system for monitoring of localized electrode nanomaterials by combining electrochemical workstation and dark-field micro/spectroscopic technology. This novel *in situ* monitoring technique achieves both the quantification and imaging of the plasmonic response that is tunable in accordance with the morphology and composition of metallic nanoparticles during the electrochemical reactions. Apparent spectral changes in intensity and wavelength with their validation via DDA simulations for model metal nanoparticles (Pt) demonstrate versatile utilities of this technique. These insights can be extended to other metallic nanoparticles and applied for rapid diagnosis of the relevant electrode materials. This monitoring platform will enable exploration of the electrochemical technology and may lead to practical sensing devices that combine high resolution and sensitivity in a simple and cost-competitive way.

## Materials and Methods

### Preparation of Au@Pt core-satellite nanoparticles

Seed-mediated growth for Au nanoparticles and diffusion-controlled reduction for Pt satellites were carried out. The detailed process is as follows:

- Seed Au nanoparticles (17 nm) were synthesized using the following procedure. HAuCl_4_∙3H_2_O (10 mg, Sigma-Aldrich) was dissolved in 100 mL of deionized water, and then the solution was heated to 100 °C with vigorous stirring. After reaching the desired temperature, 3 mL of a 1 wt% sodium citrate (Sigma-Aldrich) aqueous solution was added to the above solution and stirred for 15 min to synthesize the 17 nm Au nanoparticles. Finally, the as-obtained solution was cooled to room temperature.
- After the preparation of seed Au nanoparticles, Au nanoparticles with an average diameter of 45 nm were synthesized from the seed Au nanoparticles by using the seed-mediated growth method. HAuCl_4_∙3H_2_O (10 mg) was dissolved in 100 mL of deionized water. The solution was then heated to 100 °C with vigorous stirring. After reaching the desired temperature, 400 μL of 1 wt% sodium citrate and 4 mL of the previously obtained suspension of seed Au nanoparticles were added, and the mixture was stirred for 15 min to synthesize the 45 nm Au nanoparticles. Subsequently, the final solution was cooled to room temperature. The 45 nm Au nanoparticle plays the role of the core material in the Au@Pt core-satellite nanoparticle.
- Au@Pt core-satellite nanoparticles (active material) were then synthesized via the following procedure. The as-obtained ~45 nm Au cores (10 mL) were mixed with 10 mL of deionized water. Then, this mixture was heated to 100 °C with vigorous stirring. As a reducing agent, a specific amount of L-ascorbic acid (Aldrich) based on the desired Pt-to-Au molar ratio was added to the above solution. The molar ratio of L-ascorbic acid to H_2_PtCl_6_ was kept at 5:1 to ensure the complete reduction of the Pt precursor. Pt satellites on Au cores were then synthesized by adding a certain amount of H_2_PtCl_6_∙6H_2_O (Sigma-Aldrich) to attain a Au:Pt molar ratio of 1:1. Finally, the heating was continued for 1 h, and then the mixture was cooled to room temperature.

### Characterization of the Au@Pt assembly

The morphology was examined via high-resolution transmission electron microscopy (HR-TEM) using a JEM-3010. Atomic-distribution mapping was performed by using high angle annular dark field (HAADF)-scanning transmission electron microscopy (STEM) (JEM-2100F, 200 kV). Optical absorbance spectra were obtained via ultraviolet diffuse reflectance spectroscopy (UV-DRS, V670-Jasco).

### Preparation of electrodes

A standard three-electrode system was used with KCl-saturated Ag/AgCl as a reference electrode and a glassy carbon counter electrode. The working electrode was prepared via immobilization of Au@Pt core-satellite nanoparticles on an ITO-coated glass slide (ITO glass). Prior to the immobilization of Au@Pt core-satellites, the ITO-coated glass slide was cleaned in a mixture of organic solvents (2-propanol:acetone = 1:1 v/v) under sonication for 10 min and dried under a stream of N_2_. For the immobilization of Au@Pt core-satellite nanoparticles, 50 μL of a colloidal solution containing the as-prepared Au@Pt core-satellites was dropped onto the pretreated ITO glass. After 6 h, the electrode coated with the Au@Pt core-satellite nanoparticles was obtained.

### *in situ* measurement using the EDMC system

**Figure 1:**
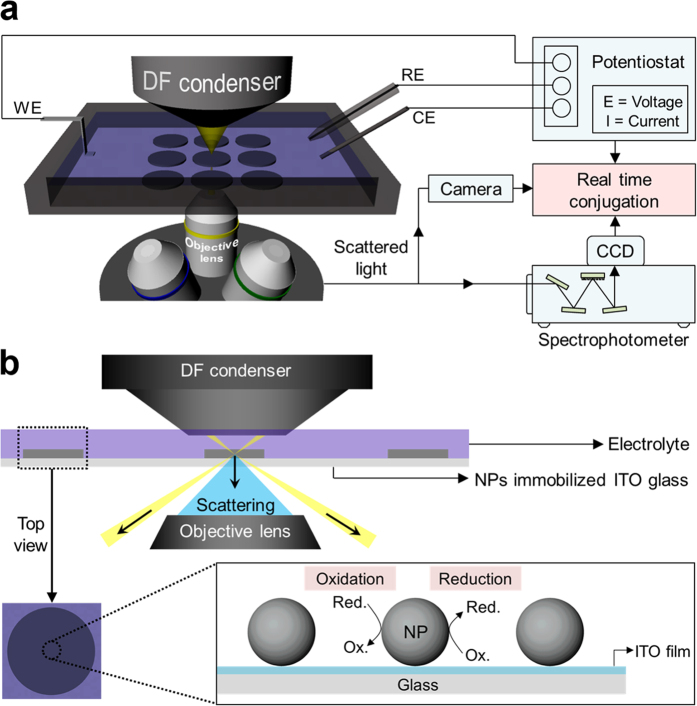
(**a**) Illustration of EDMC set-up with a dark-field microspectroscopy and the electrochemical workstation. (**b**) Schematic principle of EDMC.

**Figure 2:**
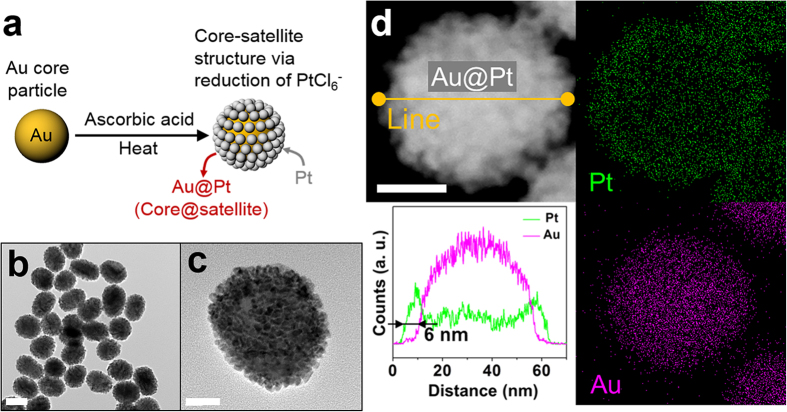
(**a**) Schematic diagram of procedure for the synthesis of Au@Pt core-satellite assembly. Au core acts as a probe for exhibiting LSPR signals during the *in situ* measurement. (**b**,**c**) High-resolution TEM images of Au@Pt core-satellites. (**d**) HAADF-STEM image of Au@Pt core-satellites particle and its elemental mapping with cross-sectional line profile. Scale bars are 50 nm (**b**) and 20 nm (**c**,**d**).

**Figure 3:**
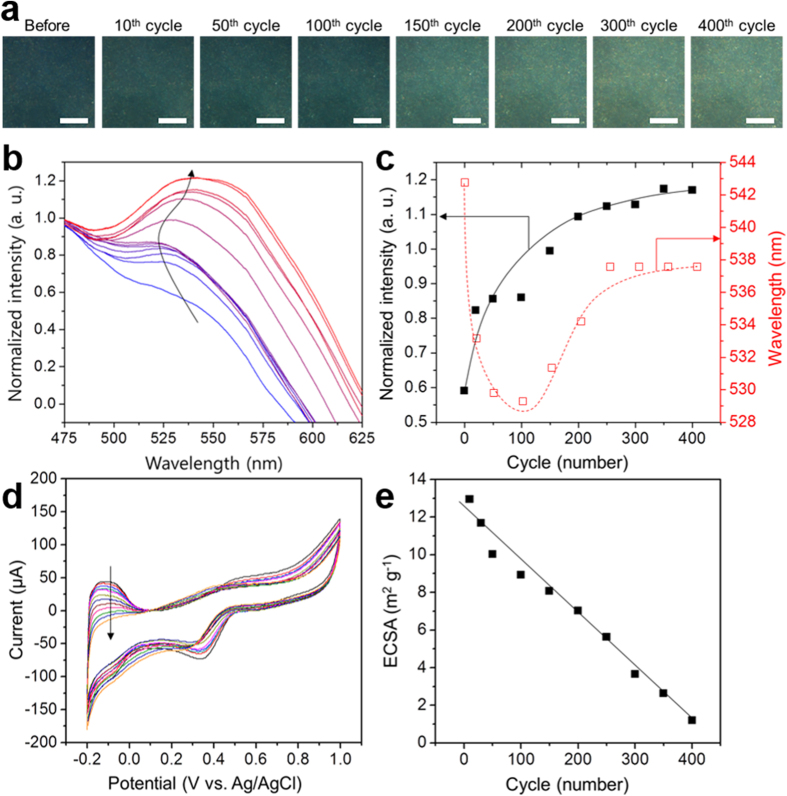
(**a**) Dark-field images of Au@Pt assembly immobilised on an ITO glass with respect to cycle number of CV. Scale bars for dark field images are 20 μm. (**b**) LSPR spectra of Au@Pt assembly with respect to cycle number of CVs. (**c)** Plots of the LSPR spectral wavelengths (λ_max_) and their intensities at λ_max_ as a function of cycle number of CVs. (**d**) CVs for Au@Pt nanoparticles during the 400 cycles with a scan rate of 30 mV sec^−1^ in 0.1 M HClO_4_. (**e**) Calculated electrochemically active surface area (ECSA) as a function of cycle number of CVs. The showing cycle numbers for (**b**) and (**d**) were 10, 20, 50, 100, 150, 200, 250, 300, 350, and 400 as the direction of the arrows.

**Figure 4:**
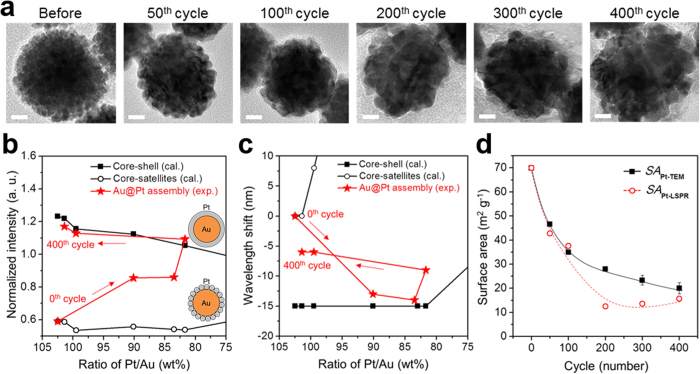
(**a**) HR-TEM images of single Au@Pt assembly showing the morphological changes with respect to the CV cycle number. Scale bars are 10 nm. (**b**) Normalized intensity and (**c**) wavelength shift of LSPR spectra obtained from DDA simulation in according to the Pt morphology and corresponding Pt/Au mass ratio (*i.e.*, cycle number). The cycle number for each remarks represent 0, 50, 100, 200, 300, and 400 in order with the direction of arrow. (**d**) The calculated total Pt surface area for Au@Pt assembly by using LSPR intensity (*SA*_Pt-LSPR_) and the TEM image (*SA*_Pt-TEM_).

The analyses were conducted in 0.1 M HClO_4_ aqueous electrolyte at room temperature. The electrolyte was purged with N_2_ for 30 min before the analyses. The cyclic voltammograms (CVs) were obtained on an electrochemical workstation (Iviumstat electrochemical analyser, Ivium Technologies). The CVs were recorded in the potential range of −0.2 to 1.0 V (vs. Ag/AgCl) at a scan rate of 30 mV s^−1^. The experiments were performed during the 400 cycles of the CVs. To monitor the LSPR spectra in accordance with the cycle number, a dark-field microscope system (Axio Observer Z1 inverted microscope) equipped with a dark-field condenser was used. As shown in Fig. 1, after the white light passes through the dark-field condenser, it is obliquely radiated onto the Au@Pt assembly nanoparticles and then only scattered light from the nanoparticles is collected by the objective lens. The control of the z-axis enables the focusing of dark field illumination. The scattering data were collected by the entrance port of a line-imaging spectrometer (Monora320i, Dongwoo Optron Co., Korea) coupled with a 1024 × 256 pixel cooled spectrograph charge-coupled device (CCD) camera (Andor Technology PLC, UK). Using this system, the light-scattering spectra can be recorded and quantified at intervals of 80 s, which is equivalent to the time consumed for 1 CV cycle. The raw optical response data were normalized with the reference light. All scattering spectra were obtained at a specific potential value of 0.5 V (vs. Ag/AgCl) during the cathodic process (1.0 V → −0.2 V, vs. Ag/AgCl) in order to exclude the effects of applied potential and the direction of sweep on the scattering characteristics (Figure S3 in the Supplementary materials)^24^.

### TEM and ICP measurement

The morphologies of the Au@Pt assembly at specific numbers of CV cycles were estimated from TEM images. The samples (*i.e.*, Au@Pt assembly nanoparticles) were collected through sonication of the working electrode before and after the 50^th^, 100^th^, 200^th^, 300^th^, and 400^th^ CV cycles. TEM images were obtained with the same particles that underwent the electrochemical reactions in the HClO_4_ electrolyte. The Pt/Au mass ratio for each cycle was examined using inductively coupled plasma atomic emission spectroscopy (ICP-AES) (JP/ICPS-7500).

### Calculation of ECSA

The electrochemically active surface area (ECSA, m^2^ g^−1^) was calculated for each cycle to generalise the standard degree of deactivation of Pt nanoparticles in this study. The ECSA can be easily calculated from the hydrogen stripping region as follows:


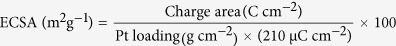


where charge area is the integrated area obtained from the hydrogen stripping peak, and the Pt loading is the amount of immobilised Pt nanoparticles.

### Discrete dipole approximation (DDA) simulation

The scattering spectra of Au@Pt core-satellites and Au@Pt core-shell nanostructures were simulated using the DDA method^25^. As depicted in the model structure of the Au@Pt core-satellites and Au@Pt core-shell (Figure S5 in the Supplementary materials), a core Au nanoparticle with a diameter of 45 nm was located at the center of the rotational coordination system. The number of satellites and the thickness of the shell were quantified using the mathematical calculations based on the size of the Au particle (diameter = 45 nm) and the experimentally obtained Pt/Au mass ratio (Table 1). Pt nanoparticles with a diameter of 4 nm were evenly distributed around the core nanoparticle at a fixed radius according to the number of satellites (1313, 1297, 1274, 1153, 1069, 1046, 952, and 414), as shown in Figure S4a (Supplementary materials). The shell thicknesses of the Au@Pt core-shells were 1.5, 2.9, 4.53, 4.62, 4.92, 5.34, 5.42, and 5.47 nm (Figure S4b in the Supplementary materials). For all DDA calculations, we used DDSCAT 7.3 code, which was developed by Drain and Flatau^23^. Approximately 20,000 dipoles were generated and used for the DDA calculation. For the calculation, the dielectric constants of Au and Ag were obtained from the literature^26^.

## Additional Information

**How to cite this article**: Song, H. D. *et al*. Real-Time Optical Monitoring of Pt Catalyst Under the Potentiodynamic Conditions. *Sci. Rep.* **6**, 38847; doi: 10.1038/srep38847 (2016).

**Publisher's note:** Springer Nature remains neutral with regard to jurisdictional claims in published maps and institutional affiliations.

## Supplementary Material

Supplementary Information

## Figures and Tables

**Table 1:** Weight ratio of Pt/Au with respect to the cycle number and the calculated surface area of Pt^a^.

# of cycle	Pt/Au (wt%)	# of Pt satellite	Surface area (nm^2^)	Thickness of Pt shell (nm)	Surface area (nm^2^)
0^th^	102.5	1313	65998.6	5.477	9835.9
50^th^	90.1	1153	57956.1	4.922	9449.5
100^th^	83.5	1069	53733.8	4.622	9243.9
200^th^	81.7	1046	52577.7	4.538	9186.7
300^th^	99.5	1274	64038.2	5.343	9741.9
400^th^	101.4	1297	65194.3	5.430	9802.8

^a^The amounts of Au were identical for all calculation. *SA*s for Pt satellites were determined using 4 nm dia. Pt spheres.
