# Supplementary material for: Real-Time Optical Monitoring of Pt Catalyst Under the Potentiodynamic Conditions
Source: Sci Rep. 2016 Dec 9;6:38847. doi: 10.1038/srep38847 (PMC5146959; doi:10.1038/srep38847)
Supplement: Supplementary Information [file srep38847-s1.pdf]

Supplementary Information

Real-Time Optical Monitoring of Pt Catalyst Under the Potentiodynamic Conditions

Hyeon Don Song[1,†], Minzae Lee[1,†], Gil-Pyo Kim[1,†], Inhee Choi[2,*], and Jongheop Yi[1,*]

[1]World Class University (WCU) Program of Chemical Convergence for Energy & Environment (C2E2), School of Chemical and Biological Engineering, Seoul National University, Gwanak-ku, Seoul 151-742, Republic of Korea.

[2]Department of Life Science, University of Seoul, Dongdaemun-ku, Seoul 130-743, Republic of Korea.

[†]These authors contributed equally to this work.

*To whom correspondence should be addressed.

E-mail:

*jyi@snu.ac.kr

*inheechoi1@uos.ac.kr

This supporting information contains:

Supplementary Figures

Supplementary Figures

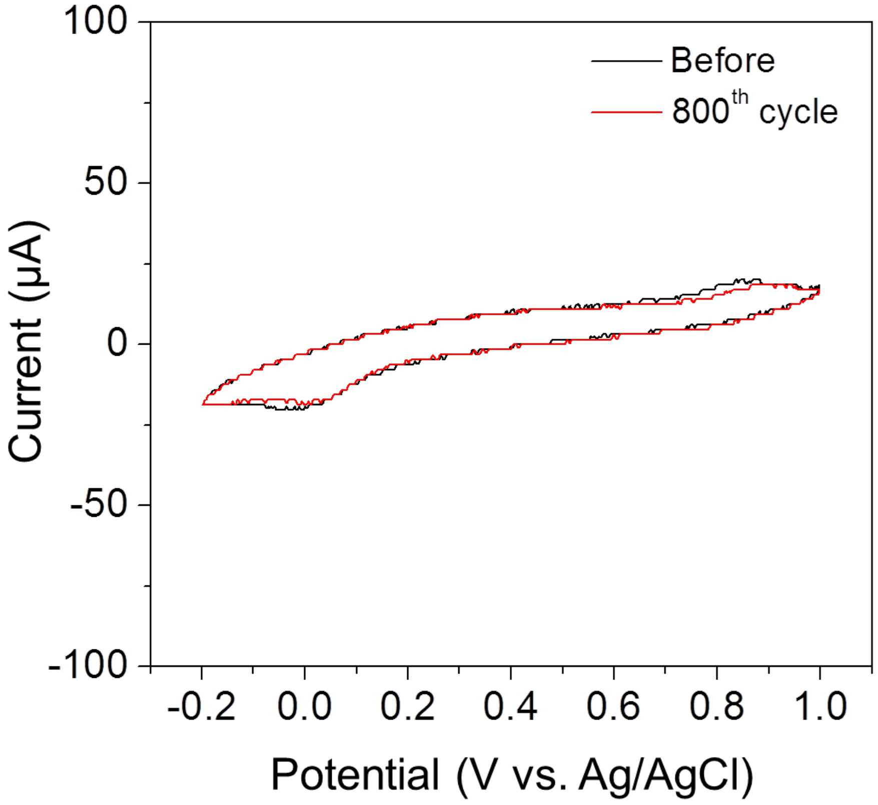

Figure S1. Cyclic voltammograms (CVs) of 45 nm Au nanoparticles at a scan rate of 30 mV sec^{-1} before and after 800 cycles. Overall shapes of CV curves were maintained as a function of number of cycles.

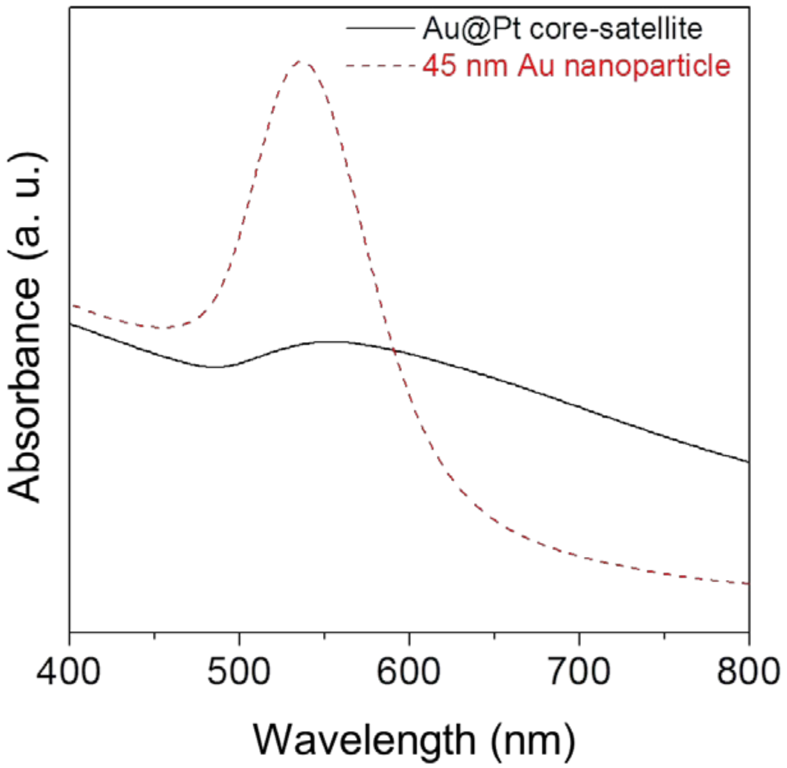

Figure S2. UV-Vis spectra for 45 nm Au nanoparticles and Au@Pt core-satellites.

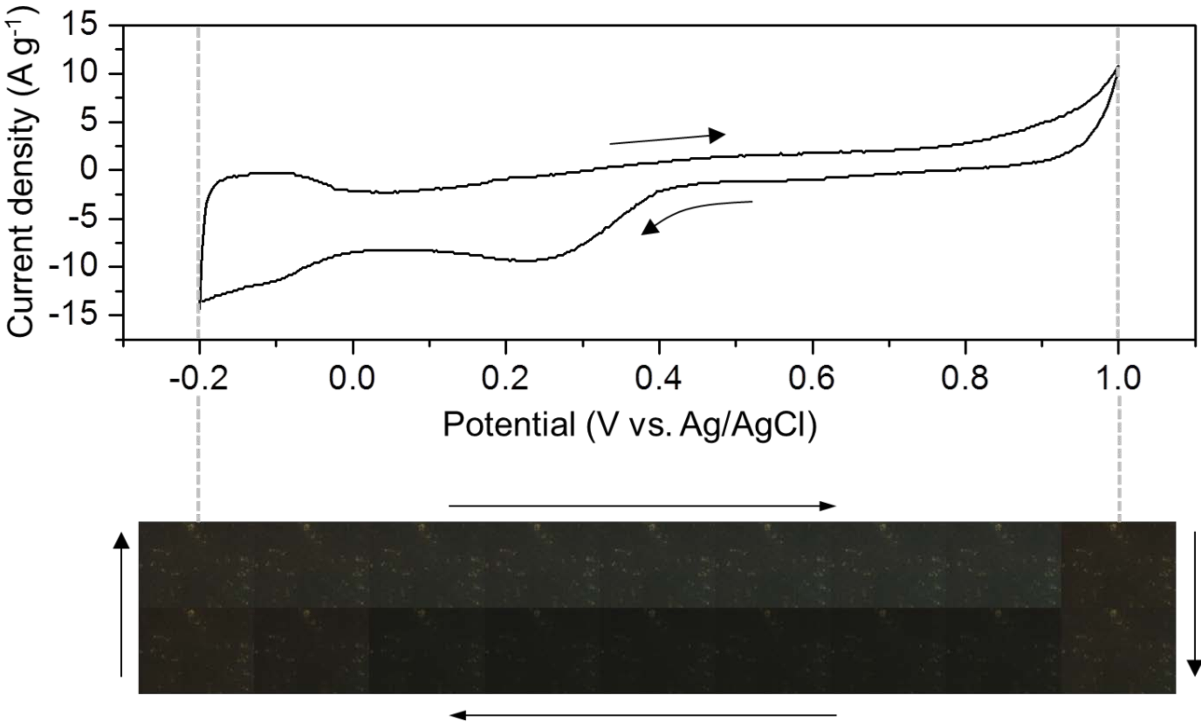

Figure S3. Changes in dark-field images of small field of view (x100) for the ITO glass during CVs. The Images were taken for one cycle.

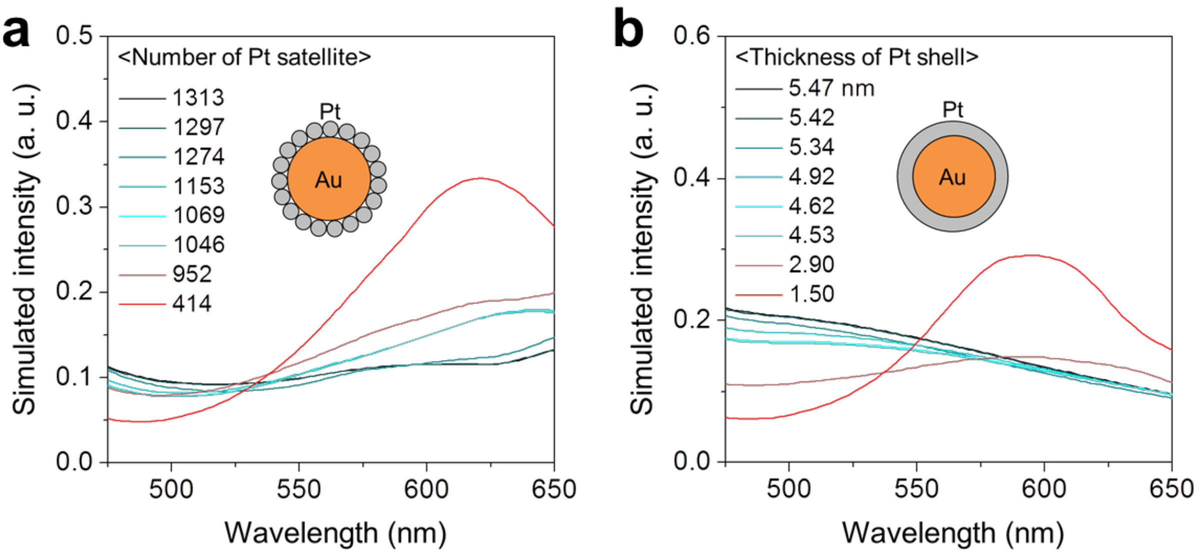

Figure S4. Theoretical LSPR spectra for (a) Au@Pt core-satellite with respect to the number of Pt satellites and (b) Au@Pt core-shell with respect to the Pt/Au ratio (wt%), which are calculated by using discrete dipole approximation (DDA) method.

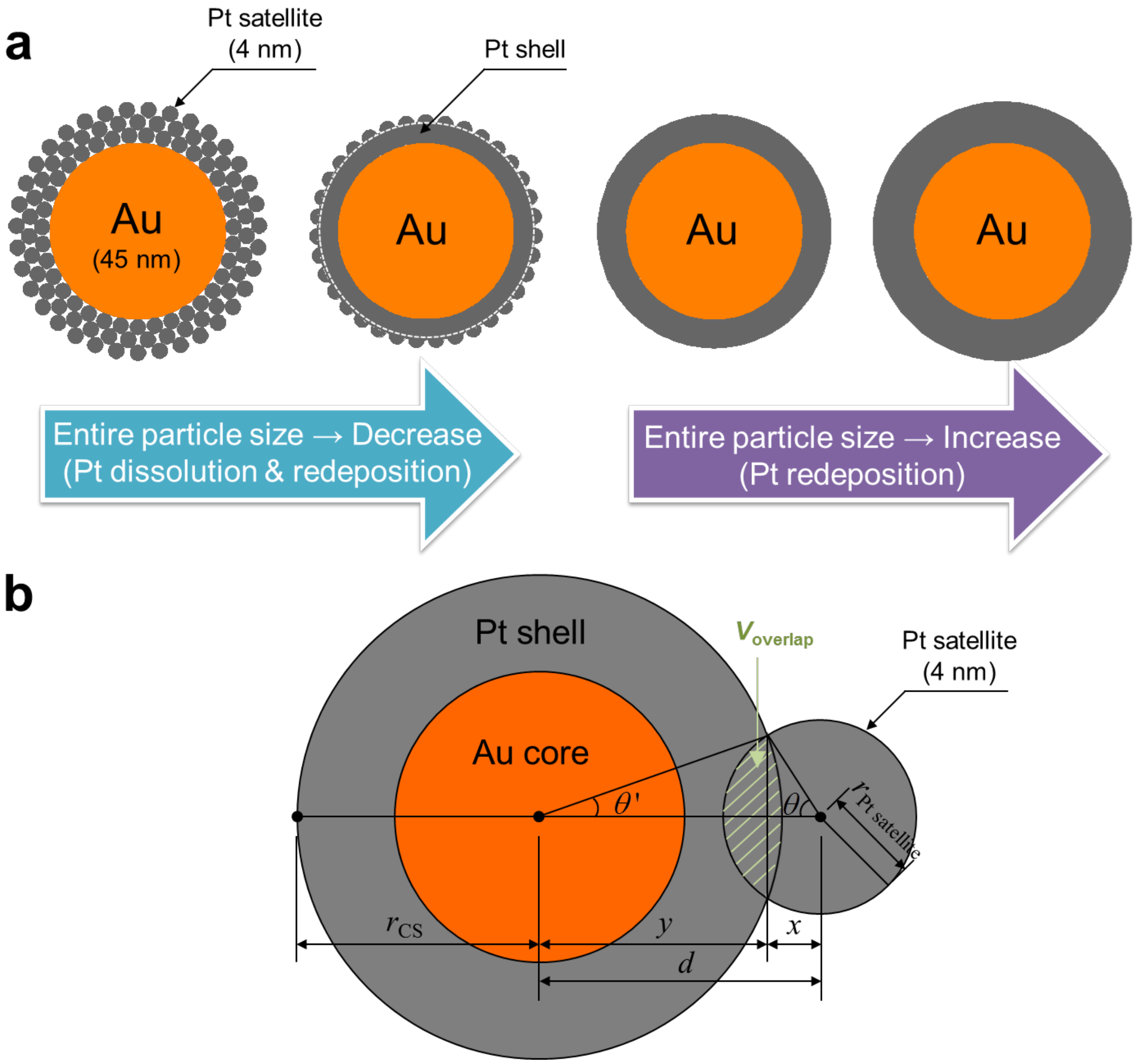

Figure S5. (a) Model morphologies of Au@Pt assembly as the increase of the CV cycle for DDA simulation. (b) Illustration of intermediate model structure with a coexistence of Pt shell and Pt satellites, showing the parameters that are considered for the calculation of the $SA_{\text{Pt-LSPR}}$.

It was assumed that the morphology transformed from the Pt satellites to the Pt shell. $SA_{\text{Pt-LSPR}}$ can be described by adopting the morphological factor *k*. The value of *k* was assumed to be 1 based on the assumption that the morphological transformation process from Au@Pt core-satellite to Au@Pt core-shell occurs under first-order kinetics. We also assumed that the overlapped Pt surface area was negligible based on following reason.

(1) The θ can be obtained by trigonometric function as follow:

$$r_{\text{Pt satellite}} - x^2 = r_{\text{CS}} - \left(d - x\right)^2$$

$$x = \frac{r_{\text{Pt satellite}}^2 - r_{CS}^2 + d^2}{2d}$$

The overlapped surface area (i.e., curved surface area) for a single Pt satellite (Single SA_{Overlap}) can be calculated by integrating its spherical surface area with $\theta = Cos^{-1}\left(\frac{x}{r_{\text{Pt satellite}}}\right)$.

Thus,

$$\text{Single } SA_{\text{Overlap}} = 2\pi r_{\text{Pt satellite}} \cdot r_{\text{Pt satellite}} \int_0^{\cos^{-1}\left(\frac{r_{\text{Pt satellite}}^2 - r_{\text{CS}}^2 + d^2}{2d \cdot r_{\text{Pt satellite}}}\right)} \sin\theta \, \mathrm{d}\theta$$

$$= 2\pi r_{\text{Pt satellite}}^2 \left(1 + \frac{r_{\text{CS}}^2 - d^2 - r_{\text{Pt satellite}}^2}{2d \cdot r_{\text{Pt satellite}}}\right)$$

Total overlapped surface area, SA_{Overlap}, is the sum of single SA_{Overlap} as follow:

$$\text{Total } SA_{\text{Overlap}} = \sum 2\pi r_{\text{Pt satellite}}^2 \left(1 + \frac{r_{\text{CS}}^2 - d^2 - r_{\text{Pt satellite}}^2}{2d \cdot r_{\text{Pt satellite}}}\right)$$

When the Pt satellites are fully surround the Au surface, it is reliable to assume that the total number of Pt satellites for a single Au@Pt core-satellite is 414 and 538 at the thickness of Pt shell ($T_{\text{Pt shell}}$)$\leq$4 nm and 4 nm$<T_{\text{Pt shell}}\leq$8 nm, respectively.

The number of Pt satellite for each case (i.e., at $T_{\text{Pt shell}}\leq$4 nm and 4 nm$<T_{\text{Pt shell}}\leq$8 nm) can also be estimated by combining with the mass ratio of Pt and the morphology factor (k).

(2) The θ' can also be obtained by trigonometric function as follow:

$$r_{CS}^2 - y^2 = r_{\text{Pt satellite}}^2 - \left(d - y\right)^2$$

$$y = \frac{r_{CS}^2 + d^2 - r_{\text{Pt satellite}}^2}{2d}$$

The overlapped volume of Pt shell can be calculated by integrating its spherical volume with $\theta' = Cos^{-1}\left(\frac{y}{r_{\text{CS}}}\right)$.

Thus,

$$\text{Single } V_{\text{Overlap}} = \pi r_{\text{Pt satellite}}^2 \cdot r_{\text{Pt satellite}} \int_0^{\cos^{-1}\left(\frac{r_{\text{Pt satellite}}^2 - r_{\text{CS}}^2 + d^2}{2d \cdot r_{\text{Pt satellite}}}\right)} \sin\theta \, \mathrm{d}\theta + \pi r_{\text{CS}}^2 \cdot r_{\text{CS}} \int_0^{\cos^{-1}\left(\frac{r_{\text{CS}}^2 + d^2 - r_{\text{Pt satellite}}^2}{2d \cdot r_{\text{CS}}}\right)} \sin\theta' \, \mathrm{d}\theta'$$

Total overlapped volume, V_{Overlap}, can be calculated by the summation of overlapped volume of Pt shell and the overlapped volume of Pt satellites as follow:

$$\text{Total } SA_{\text{Overlap, V}} = \sum \frac{3}{r_{\text{Pt satellite}}} \left\{ \pi r_{\text{Pt satellite}}^3 \left(1 + \frac{r_{\text{CS}}^2 - d^2 - r_{\text{Pt satellite}}^2}{2d \cdot r_{\text{Pt satellite}}} \right) + \pi r_{\text{CS}}^3 \left(1 + \frac{r_{\text{Pt satellite}}^2 - d^2 - r_{\text{CS}}^2}{2d \cdot r_{\text{CS}}} \right) \right\}$$

Then, the total overlapped volume of Pt can be converted into the total surface area of Pt caused by overlapped volume, $SA_{\text{Overlap, V}}$.

With considering that $r_{\text{Pt satellite}}$ is 2 nm, $\text{Total } SA_{\text{Overlap, V}} - \text{Total } SA_{\text{Overlap}} << SA_{\text{COSA}} + SA_{\text{CS}}$

Thus, it is reasonable to assume that the overlapped Pt surface area is negligible.

Thus, $SA_{\text{Pt-LSPR}}$ can be described as $SA_{\text{Pt-LSPR}} = f(k, V_{\text{Pt}})$. $SA_{\text{Pt-LSPR}}$ is the sum of the surface areas obtained from the Pt satellites (SA_{COSA}) and Pt shell (SA_{CS}) through the following equations:

$$SA_{\text{Pt-LSPR}} = SA_{\text{COSA}} + SA_{\text{CS}}$$

$$SA_{\text{COSA}} = \frac{3V_{\text{COSA}}}{2} = \frac{3\left(V_{\text{Pt}} - kV_{\text{Pt}}\right)}{2}$$

$$SA_{\text{CS}} = 4\pi \left\{ \left(r_{\text{Au}}\right)^3 + \frac{3kV_{\text{Pt}}}{4\pi} \right\}^{2/3}$$

where V_{COSA} is the volume of Pt in the Au@Pt core-satellite, V_{Pt} is the total volume of Pt, r_{Au} is the radius of a Au core nanoparticle (22.5 nm), and d is the distance from the center of a Au core particle to the center of each Pt satellite. The densities of Pt (21.45 g cm^{-3}) and Au (19.30 g cm^{-3}) were used for the calculation.

$SA_{\text{Pt-TEM}}$ can be calculated using the following equation:

$$SA_{\text{Pt-TEM}} = V_{\text{Pt}} \times \frac{4\pi r_{\text{Pt satellite}}^2}{4/3\, \pi r_{\text{Pt satellite}}^3} = \frac{3V_{\text{Pt}}}{r_{\text{Pt satellite}}}$$

where r_{Pt} satellite is the radius of the Pt satellite measured from the TEM images. The measured values of r_{Pt} satellite are presented as following figure.

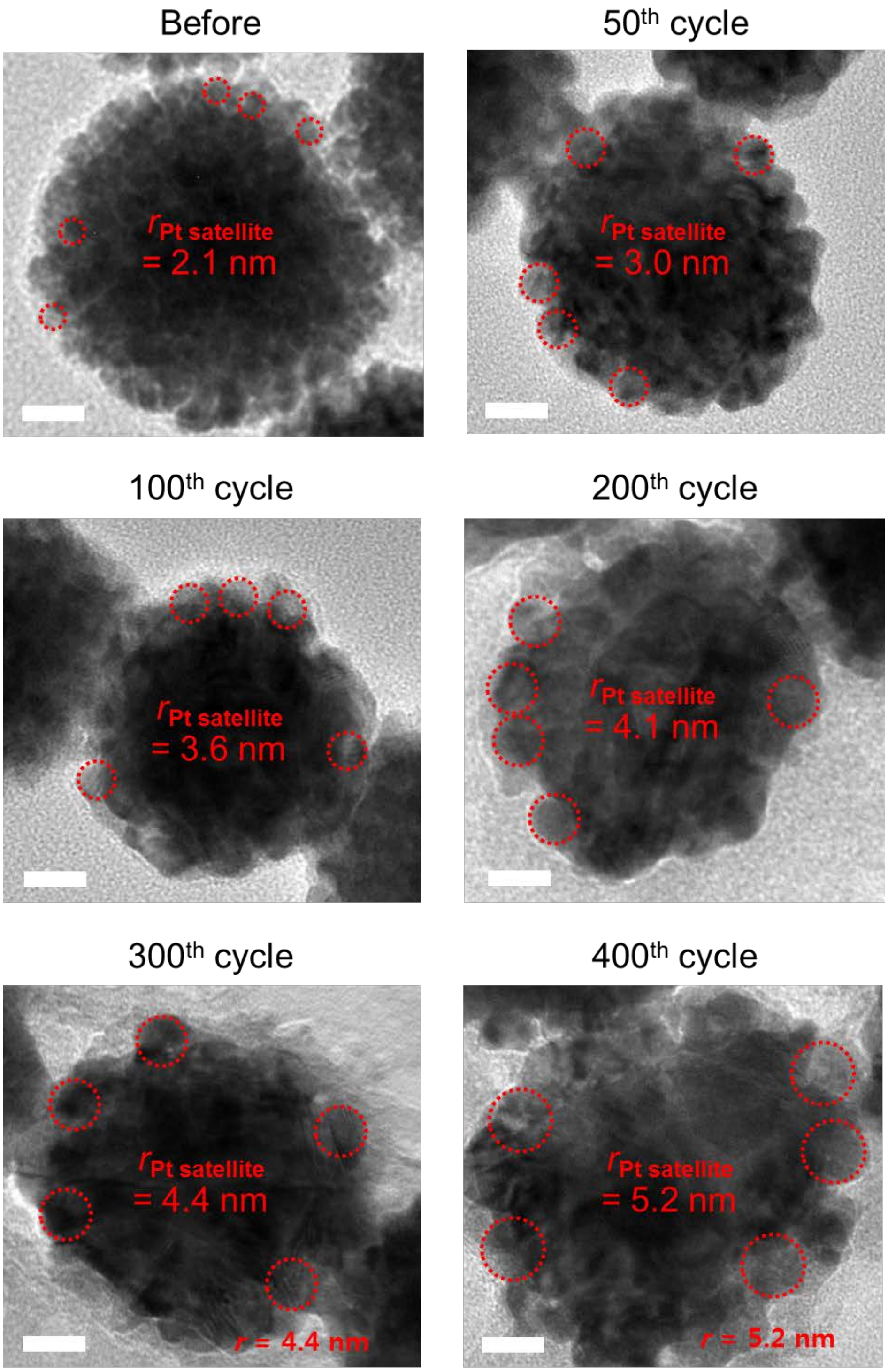

The TEM images were obtained after each CV cycle. The $r_{Pt\ satellite}$ is the average value of five representatives. Scale bars for the TEM images are 10 nm.
